# Compression-based classification of biological sequences and structures via the Universal Similarity Metric: experimental assessment

**DOI:** 10.1186/1471-2105-8-252

**Published:** 2007-07-13

**Authors:** Paolo Ferragina, Raffaele Giancarlo, Valentina Greco, Giovanni Manzini, Gabriel Valiente

**Affiliations:** 1 Dipartimento di Informatica, Università di Pisa, Italy; 2 Dipartimento di Matematica Applicazioni, Università di Palermo, Italy; 3 Dipartimento di Informatica, Università del Piemonte Orientale, Italy; 4 Algorithms, Bioinformatics, Complexity and Formal Methods Research Group, Technical University of Catalonia, Barcelona, Spain

## Abstract

**Background:**

Similarity of sequences is a key mathematical notion for Classification and Phylogenetic studies in Biology. It is currently primarily handled using alignments. However, the alignment methods seem inadequate for post-genomic studies since they do not scale well with data set size and they seem to be confined only to genomic and proteomic sequences. Therefore, alignment-free similarity measures are actively pursued. Among those, USM (Universal Similarity Metric) has gained prominence. It is based on the deep theory of Kolmogorov Complexity and *universality *is its most novel striking feature. Since it can only be approximated via data compression, USM is a methodology rather than a formula quantifying the similarity of two strings. Three approximations of USM are available, namely UCD (Universal Compression Dissimilarity), NCD (Normalized Compression Dissimilarity) and CD (Compression Dissimilarity). Their applicability and robustness is tested on various data sets yielding a first massive quantitative estimate that the USM methodology and its approximations are of value. Despite the rich theory developed around USM, its experimental assessment has limitations: only a few data compressors have been tested in conjunction with USM and mostly at a qualitative level, no comparison among UCD, NCD and CD is available and no comparison of USM with existing methods, both based on alignments and not, seems to be available.

**Results:**

We experimentally test the USM methodology by using 25 compressors, all three of its known approximations and six data sets of relevance to Molecular Biology. This offers the first systematic and quantitative experimental assessment of this methodology, that naturally complements the many theoretical and the preliminary experimental results available. Moreover, we compare the USM methodology both with methods based on alignments and not. We may group our experiments into two sets. The first one, performed via ROC (Receiver Operating Curve) analysis, aims at assessing the *intrinsic *ability of the methodology to discriminate and classify biological sequences and structures. A second set of experiments aims at assessing how well two commonly available classification algorithms, UPGMA (Unweighted Pair Group Method with Arithmetic Mean) and NJ (Neighbor Joining), can use the methodology to perform their task, their performance being evaluated against gold standards and with the use of well known statistical indexes, i.e., the F-measure and the partition distance. Based on the experiments, several conclusions can be drawn and, from them, novel valuable guidelines for the use of USM on biological data. The main ones are reported next.

**Conclusion:**

UCD and NCD are indistinguishable, i.e., they yield nearly the same values of the statistical indexes we have used, accross experiments and data sets, while CD is almost always worse than both. UPGMA seems to yield better classification results with respect to NJ, i.e., better values of the statistical indexes (10% difference or above), on a substantial fraction of experiments, compressors and USM approximation choices. The compression program PPMd, based on PPM (Prediction by Partial Matching), for generic data and Gencompress for DNA, are the best performers among the compression algorithms we have used, although the difference in performance, as measured by statistical indexes, between them and the other algorithms depends critically on the data set and may not be as large as expected. PPMd used with UCD or NCD and UPGMA, on sequence data is very close, although worse, in performance with the alignment methods (less than 2% difference on the F-measure). Yet, it scales well with data set size and it can work on data other than sequences. In summary, our quantitative analysis naturally complements the rich theory behind USM and supports the conclusion that the methodology is worth using because of its robustness, flexibility, scalability, and competitiveness with existing techniques. In particular, the methodology applies to all biological data in textual format. The software and data sets are available under the GNU GPL at the supplementary material web page.

## Background

The notion of distance and similarity between two strings is a very important and widely studied one [1-4] since it plays a fundamental role in biological sequence analysis, phylogeny and classification. Classically, those notions hinge on sequence alignment methods. However, distance and similarity functions based on alignment methods do not scale well with data set size and they are no longer perceived as adequate now that entire genomes are available [5]. Moreover, they are not flexible, since they can only be used with genomic and proteomic sequences. Therefore, novel alignment-free functions are actively pursued, the ones based on textual compression being natural candidates because of the deep connection of compression with classification and modeling [6].

In this scenario, Li et al. [7] have devised a *universal *similarity metric for strings-a remarkable achievement since universality means it is a lower bound for all the computable distance and similarity functions, including all the ones so far considered for biological applications, e.g., [2-5]. Unfortunately, being the measure based on Kolmogorov complexity [8], it is not computable. However, one can still get a practical tool from such a beautiful theoretical finding since the Kolmogorov complexity of a string can be approximated via data compression [8]. This leaves open the problem of how to best approximate the universal measure, which can be regarded more as a methodology than a formula quantifying how similar two objects are. Three distinct functions have been proposed as an approximation to USM: UCD, NCD and CD. We point out that two of them have been slightly changed in this work, with respect to their first appearance in the literature, to make our study consistent. Moreover, USM is a distance function (despite its name) implying that its three approximations are dissimilarity functions. In turn, the discriminative abilities of UCD, NCD and CD depend critically on the compression algorithm one uses for their computation. NCD has been the object of deep theoretical studies in [7,9], where experimental evidence of its validity has also been initially assessed. CD has been used for classification and data mining in [10] and it was obtained independently in [11,12] in the realm of table compression. UCD has been used in [13-15] to classify protein structures. Those studies, although groundbreaking, seem to be only an initial assessment of the power of the new methodology and leave open fundamental experimental questions that need to be addressed in order to establish how appropriate the use of the methodology is for classification of biological data. The main ones are the following, which should be addressed accross data sets of biological relevance and with the aid of well known statistical indexes to quantify performance: (A) how well does the methodology classify and, in particular, which of UCD, NCD and CD is the best performer; (B) which classification algorithm performs best when using the methodology; (C) How does the classification ability of the formulas depend on the choice of compression programs, i.e., the experiments conducted so far exclude weak compression programs such as memoryless ones because they are likely to give bad results, yet they are very fast to be outright dismissed; (D) how does the methodology compare with existing methods, both based on alignments and not, i.e., whether it is worthy of consideration simply because it scales well with data set size and it can work on data other than genomic or proteomic sequences, or because it is also competitive even on data sets that could be analyzed with alternative methods, by now standard.

We provide two sets of experiments designed to shed light on the mentioned problems, contributing the first substantial experimental assessment of USM, of its possible uses in Molecular Biology and naturally complementing both the theory and the initial experimental work done so far to sustain USM.

## Results and discussion

### Experimental setup

Several benchmark data sets of non-homologous protein structures have been developed in the last few years [16-19]. In this study, we have chosen the 36 protein domains of [16] and the 86 prototype protein domains of [17]. The Chew-Kedem data set, which consists of 36 protein domains drawn from PDB entries of three classes (alpha-beta, mainly-alpha, mainly-beta), was introduced in [16] and further studied in [13]. The Sierk-Pearson data set, which consists of a non-redundant subset of 2771 protein families and 86 non-homologous protein families from the CATH protein domain database [20], was introduced in [17].

**Figure 1:**
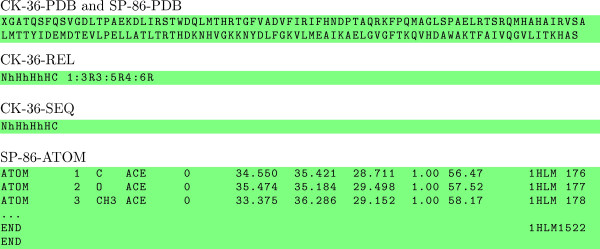
**Alternative representations**. PDB protein domain 1hlm00, a globin from the sea cucumber *Caudina arenicola*, the only protein common to the Chew-Kedem data set (CK-36-PDB and SP-86-PDB: amino acid sequence in FASTA format; CK-36-REL: complete TOPS string, with contact map; CK-36-SEQ: TOPS string of secondary structure elements) and Sierk-Pearson data set (SP-86-ATOM: ATOM lines from the PDB entry).

For both the Chew-Kedem and the Sierk-Pearson data sets, we have considered several alternative representations. Besides the standard representation of amino acid sequences in FASTA format [21], we have also used a text file consisting of the ATOM lines in the PDB entry for the protein domain, the topological description of the protein domain as a TOPS string of secondary structure elements [22-25], and the complete TOPS string with the contact map. The TOPS model is based on secondary structure elements derived using DSSP [26], plus the chirality algorithm of [25]. For instance, the various representations of PDB protein domain 1hlm00, a globin from the sea cucumber *Caudina arenicola *[27,28], are illustrated in Figure 1.

We have also included in this study a benchmark data set of 15 complete unaligned mitochondrial genomes, referred to as the Apostolico data set [29].

In summary, the six data sets with the acronyms used in this paper are as follows:

AA-15-DNA: Apostolico data set of 15 species, mitochondrial DNA complete genomes.

CK-36-PDB: Chew-Kedem data set of 36 protein domains, amino acid sequences in FASTA format.

CK-36-REL: Chew-Kedem data set of 36 protein domains, complete TOPS strings with contact map.

CK-36-SEQ: Chew-Kedem data set of 36 protein domains, TOPS strings of secondary structure elements.

SP-86-ATOM: Sierk-Pearson data set of 86 protein domains, ATOM lines from PDB entries.

SP-86-PDB Sierk-Pearson data set of 86 protein domains, amino acid sequences.

We considered twenty different compression algorithms and, for some of them, we tested up to three variants. The choice of the compression algorithms reflects quite well the spectrum of data compressors available today, as outlined in the Methods section. Finally, dissimilarity matrices, both corresponding to the USM methodology and to other well established techniques, were computed as described in the Methods section.

### Intrinsic classification abilities: the ROC analysis

This set of experiments aims at establishing the intrinsic classification ability of the dissimilarity matrices obtained via each data compressor and each of UCD, NCD and CD. In order to measure how well each dissimilarity matrix separates the objects in the data set at the level of CATH class, architecture, and topology, we have taken the similarity of two protein domains as the score of a binary classifier putting them into the same class, architecture, or topology, as follows.

We first converted each of the 36 × 36 dissimilarity matrices (for the CK-36 data set) and each of the 86 × 86 dissimilarity matrices (for the SP-86 data set) to *similarity *vectors of length 1,296 and 7,398, respectively, and used each of these vectors, in turn, as predictions. Also, for each classification task, we obtained a corresponding symmetric matrix with entries 1 if the two protein domains belong to the same CATH class, architecture, or topology (depending on the classification task) and 0 otherwise, and we converted these matrices to vectors of length 1,296 and 7,398, respectively, and used them as class labels. We have performed the ROC analysis using the ROCR package [30]. The relevant features about ROC analysis are provided in the Methods section.

**Figure 2:**
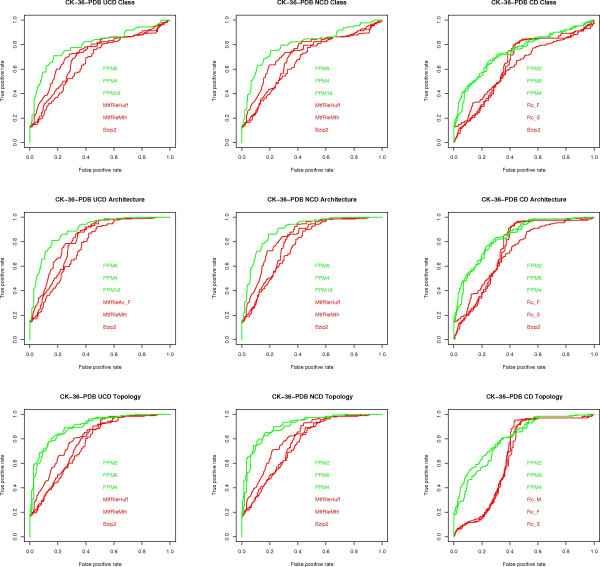
**ROC curves for CK-36-PDB**. ROC curves for the CK-36-PDB data set, one for each classification task (class, architecture, topology) and each measure (UCD, NCD, CD). Only the three algorithms with highest (green) and lowest (red) AUC values are shown.

**Figure 3:**
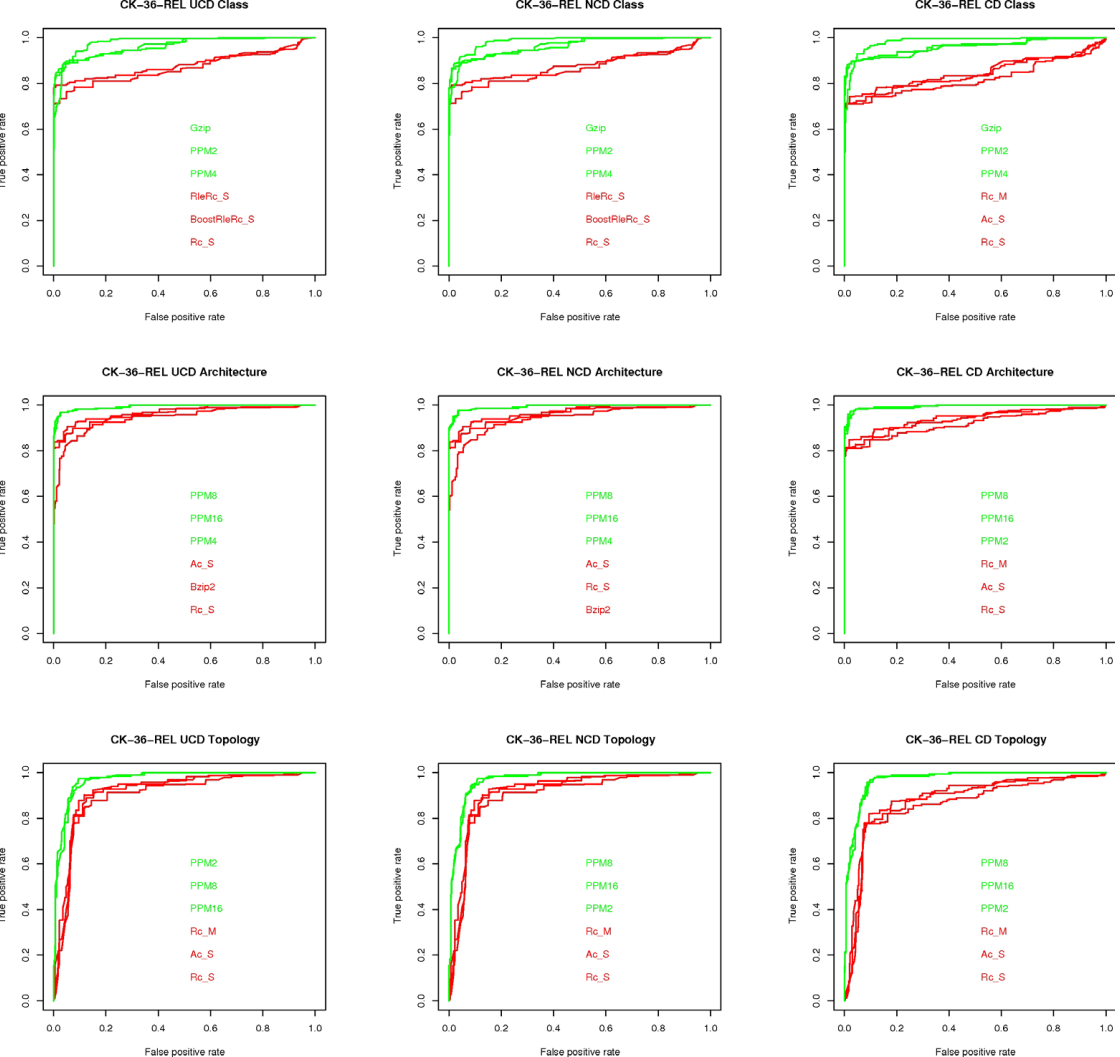
**ROC curves for CK-36-REL**. ROC curves for the CK-36-REL data set, one for each classification task (class, architecture, topology) and each measure (UCD, NCD, CD). Only the three algorithms with highest (green) and lowest (red) AUC values are shown.

**Figure 4:**
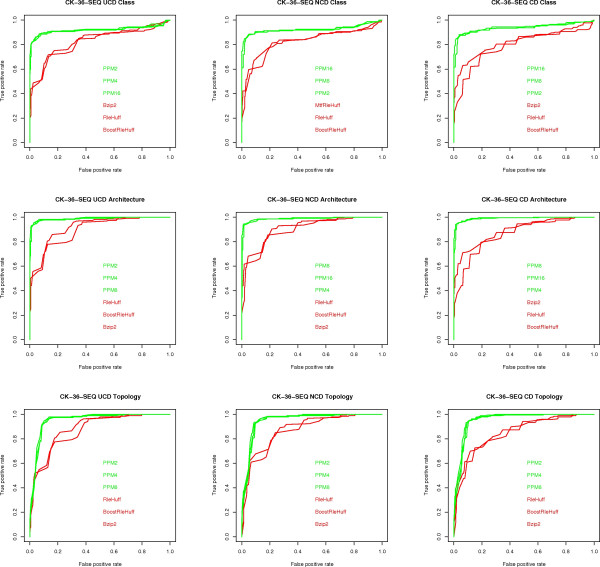
**ROC curves for CK-36-SEQ**. ROC curves for the CK-36-SEQ data set, one for each classification task (class, architecture, topology) and each measure (UCD, NCD, CD). Only the three algorithms with highest (green) and lowest (red) AUC values are shown.

**Figure 5:**
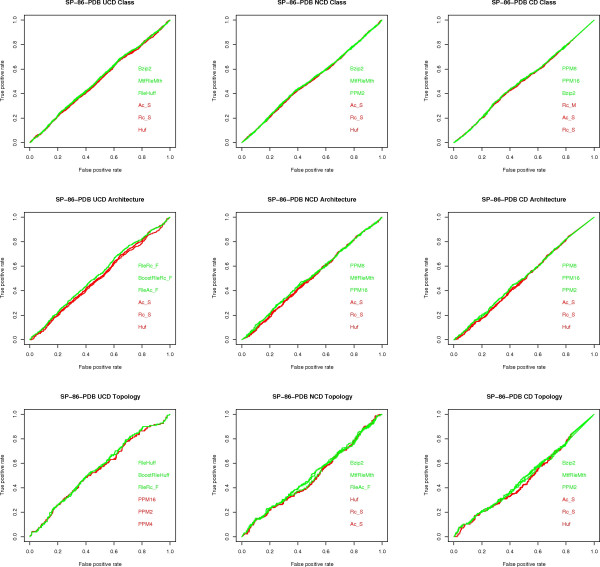
**ROC curves for SP-86-PDB**. ROC curves for the SP-86-PDB data set, one for each classification task (class, architecture, topology) and each measure (UCD, NCD, CD). Only the three algorithms with highest (green) and lowest (red) AUC values are shown.

**Figure 6:**
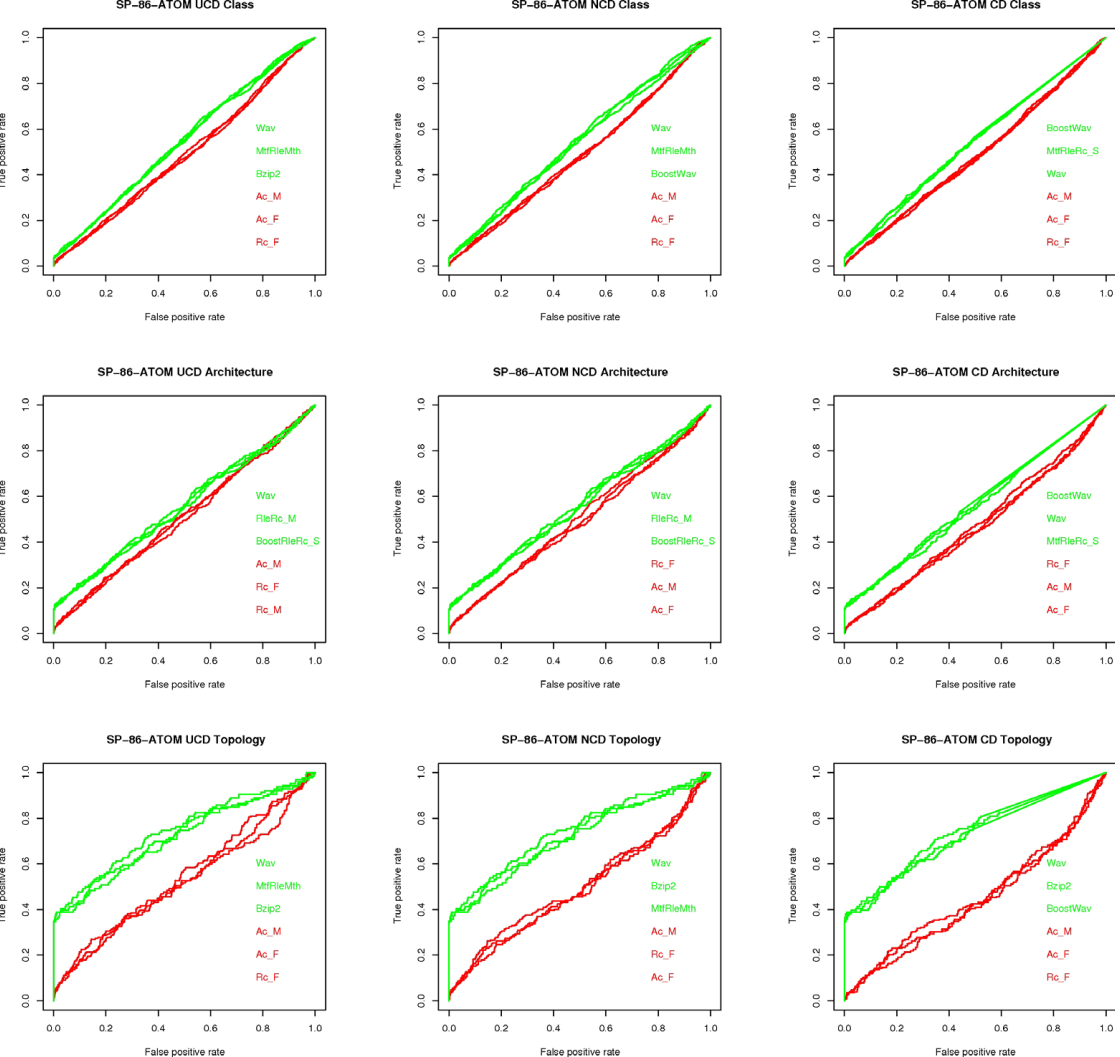
**ROC curves for SP-86-ATOM**. ROC curves for the SP-86-ATOM data set, one for each classification task (class, architecture, topology) and each measure (UCD, NCD, CD). Only the three algorithms with highest (green) and lowest (red) AUC values are shown.

The result of these experiments is a total of 5 × 3 × 3 × 24 = 1, 080 AUC (Area under the ROC curve) values, one for each of the five alternative representations of the CK-36 and SP-86 data sets, three dissimilarity measures, three classification tasks, and 25 compression algorithms, together with 5 × 3 × 3 = 45 ROC plots, which are summarized in Figures 2, 3, 4, 5, 6.

**Figure 7:**
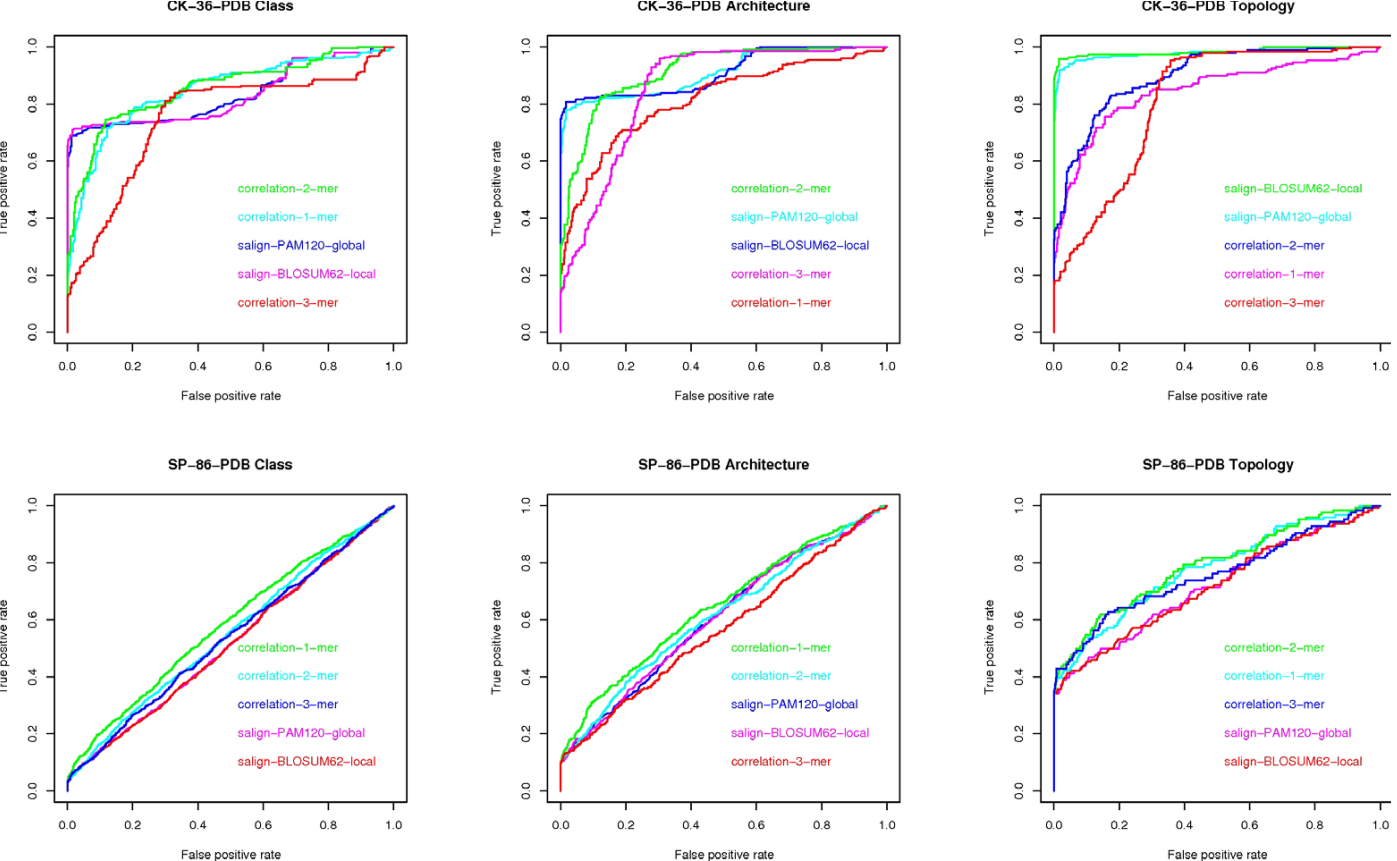
**ROC curves for alignment and *k*-mer frequencies**. ROC curves for global and local alignment and *k*-mer frequencies, one for each data set (CK-36-PDB and SP-86-PDB) and each classification task (class, architecture, topology).

The following conclusions can be drawn from the experiments, with reference to the open questions posed in the Background section:

1) **Question (A)**. UCD and NCD are virtually indistinguishable and have an AUC value at least as good as that of CD in most cases. That is, once we have fixed the data set, classification task, and compressor, the value of the AUC index is quite close for UCD and NCD and, in most cases, better than CD. In terms of the disciminative power of UCD, NCD and CD, the top performing compression algorithms, e.g., PPMd, achieve an AUC value ranging from a minimum of 0.80 to a maximum of 0.96 for the various classification tasks on the various representations of the CK-36 proteins. These are excellent values, given that a perfect classification has an AUC score of 1. For the SK-86 data set, all measures performed poorly on all classification tasks. Since neither the alignment methods nor the alignment-free methods did better, this is an indication that the data set is hard to classify.

2) **Question (C) **The PPMd compressors are consistently at the top of the AUC index. Moreover, Gzip provides in general a comparable performance: a maximum 8% difference in AUC values, although in most cases the difference is much smaller and in one case Gzip is better. On the other hand, Bzip2 is lagging behind: a 17% maximum difference in AUC values. The difference between "memoryless" and "with memory" compressors is rather subtle. As already said for the SK-86 data set, all measures did poorly, across compressors, classification tasks and data representation. For the CK-36 data set, "with memory" compressors had a noticeable gain in performance, across classification tasks, only on CK-36-PDB: a 15% maximum difference in AUC values. As for REL and SEQ, the difference in performance is a maximum 7% difference in AUC values, although most compressors are quite close to the maximum AUC values.

3) **Question (D) **Here we consider the maximum AUC value given by the existing methods (see fig 7) versus the maximum AUC value given by the compression based measures. The difference is in favor of the former methods by 2% on architecture, 4% on class and 9% on topology, on the CK-36-PDB data set. On the SK-86-PDB data set, none of the methods performed very well, i.e., all AUC values were below 0.70. This seems to indicate that the main advantage of the USM methodology is its scalability with data set size.

### Classification via algorithms: UPGMA and NJ

This set of experiments aims at establishing how well the dissimilarity matrices computed via UCD, NCD and CD, with different compressors, can be used for classification by two well known and widely available classification algorithms. We have chosen UPGMA [31] and NJ [32], two classic tree construction algorithms, as implemented in BioPerl [33]. In relation to the clustering literature [34], both can be considered as Hierarchical Methods. In fact, UPGMA is also known as Average Link Hierarchical Clustering. NJ does not seem to have a counterpart in the clustering literature, although it certainly belongs to the Hierarchical Clustering algorithmic paradigm.

In order to assess the performance of compression-based classification, via UPGMA and NJ under various compression algorithms, we have computed two external measures [34], the *F*-measure and the partition distance, against a gold standard. The relevant features of those two measures is presented in the Methods section.

For the classification of protein domains, we have taken as the gold standard the CATH classification [20], although we might have adopted the SCOP classification [35] instead and, as a matter of fact, there are ongoing efforts to combine both classifications of protein domains [36,37]. In order to obtain a partitional clustering solution from the tree computed by UPGMA, we place in the same cluster all proteins that descend from the same level one ancestor in the UPGMA tree. Then, we can compute the F-measure by using this clustering solution and the gold standard division of proteins in groups according to CATH class. The same procedure is used for NJ.

On the other hand, the classification of species we have taken as the gold standard is the NCBI taxonomy [38]. In this case, we can simply compare the two trees computed by UPGMA and NJ against the NCBI taxonomy, via the partition distance. Again, we might have adopted any other classification of species instead such as, for instance, the global phylogeny of fully sequenced organisms of [39].

We first computed for each compression algorithm the UCD, NCD and CD dissimilarity measures over all pairs of elements in the data set, then obtained classification trees using UPGMA and NJ and finally, computed the *F*-measure between the clustering solution obtained from those classification trees and the corresponding gold standard, for the first five data sets. We used the partition distance for the last one, as mentioned already. We followed an analogous procedure in order to obtain results via the non-compressive methods, the only difference being the computation of the dissimilarity matrix. Tables 1, 2, 3, 4, 5, 6 report the results on the six different data sets for the compression based measures, whereas Table 7 summarizes the ones obtained using the standard methods.

**Table 1:** CK-36-PDB

CK-36-PDB	UCD		NCD		CD	
	
	UPGMA	NJ	UPGMA	NJ	UPGMA	NJ
Gzip	0.7665	0.7454	0.8196	0.7603	0.7360	0.7000
Bzip2	0.7872	0.7069	0.7656	0.7130	0.7452	0.6685
PPMd16	0.9605	0.8072	0.9605	0.9024	0.7850	0.7403
PPMd8	0.9605	0.8072	0.9605	0.9024	0.9030	0.7820
PPMd4	0.9605	0.8146	0.9605	0.9024	0.9030	0.7820
PPMd2	0.9351	0.8072	0.9420	0.7603	0.8881	0.7450
Huffman	0.8004	0.7224	0.8004	0.7224	0.7541	0.7233
Ac fast	0.8274	0.7419	0.8274	0.7419	0.7541	0.7362
Rc fast	0.8216	0.7308	0.8004	0.7308	0.7708	0.7691
Ac med.	0.8004	0.7276	0.8274	0.7276	0.7611	0.8111
Rc med.	0.8274	0.7276	0.8447	0.7234	0.7708	0.7223
Ac slow	0.8447	0.7331	0.8274	0.7331	0.7708	0.7223
Rc slow	0.8274	0.7331	0.8447	0.7276	0.7708	0.7747
BwtRleHuff	0.8666	0.7789	0.8778	0.7789	0.7950	0.7609
BwtMtfRleHuff	0.7850	0.7577	0.7950	0.7773	0.7625	0.7424
BwtRleAc fast	0.7944	0.7677	0.7944	0.7677	0.7850	0.7218
BwtMtfRleAc fast	0.8045	0.8046	0.8320	0.7577	0.7452	0.7019
BwtRleRc fast	0.8778	0.7677	0.8778	0.7677	0.7804	0.7505
BwtMtfRleRc fast	0.8309	0.8046	0.8309	0.8046	0.7619	0.7172
BwtRleRc med.	0.8778	0.7789	0.8778	0.7789	0.7950	0.7655
BwtMtfRleRc med.	0.8347	0.8046	0.8135	0.7577	0.7619	0.6970
BwtRleRc slow	0.8666	0.7677	0.8666	0.7603	0.7850	0.7933
BwtMtfRleRc slow	0.8420	0.7503	0.8235	0.7503	0.7619	0.6970
BwtWavelet	0.8497	0.9186	0.8497	0.8281	0.7486	0.6970

Experimental results for the CK-36-PDB data set, with the UCD (left), NCD (middle), and CD (right) distance. For each compression algorithm, we report the *F*-measure for both UPGMA and NJ methods. The F-measure is a value ranging from 0 for highest dissimilarity to 1 for identical classifications.

**Table 2:** CK-36-REL

CK-36-REL	UCD		NCD		CD	
	
	UPGMA	NJ	UPGMA	NJ	UPGMA	NJ
Gzip	0.8870	0.7820	0.8870	0.8676	0.9400	0.8195
Bzip2	0.9030	0.8197	0.9030	0.7970	0.9030	0.8096
PPMd16	0.9030	0.9030	0.9030	0.7820	0.9030	0.9015
PPMd8	0.9030	0.7820	0.9030	0.7820	0.9030	0.7600
PPMd4	0.9030	0.7820	0.9030	0.7820	0.9030	0.8478
PPMd2	0.8881	0.7450	0.9030	0.8329	0.9030	0.7600
Huffman	0.8663	0.7700	0.8663	0.7700	0.8274	0.7658
Ac fast	0.8580	0.7639	0.8580	0.7639	0.9030	0.7777
Rc fast	0.8580	0.7639	0.8400	0.7820	0.8734	0.7288
Ac med.	0.8881	0.7820	0.8881	0.7820	0.8734	0.7777
Rc med.	0.8580	0.7440	0.8580	0.7820	0.8274	0.8212
Ac slow	0.8699	0.7820	0.8699	0.7820	0.8022	0.7658
Rc slow	0.8706	0.8706	0.8706	0.7440	0.7552	0.7658
BwtRleHuff	0.8706	0.7558	0.8706	0.7440	0.9030	0.7694
BwtMtfRleHuff	0.8500	0.8542	0.8518	0.7558	0.8706	0.7275
BwtRleAc fast	0.8881	0.7440	0.8881	0.7820	0.9030	0.7694
BwtMtfRleAc fast	0.8881	0.8542	0.8881	0.8850	0.9030	0.7943
BwtRleRc fast	0.8518	0.8923	0.8518	0.8542	0.9030	0.7694
BwtMtfRleRc fast	0.8706	0.8188	0.8706	0.8146	0.9030	0.7718
BwtRleRc med.	0.8500	0.7440	0.8500	0.7440	0.8706	0.7970
BwtMtfRleRc med.	0.8518	0.7558	0.8706	0.8188	0.9030	0.8863
BwtRleRc slow	0.8881	0.7440	0.8881	0.7398	0.8500	0.7694
BwtMtfRleRc slow	0.8881	0.7558	0.8881	0.8542	0.8500	0.7690
BwtWavelet	0.8392	0.9084	0.8400	0.7898	0.8458	0.7288

Experimental results for the CK-36-REL data set, with the UCD (left), NCD (middle), and CD (right) distance. For each compression algorithm, we report the F-measure for both UPGMA and NJ methods.

**Table 3:** CK-36-SEQ

CK-36-SEQ	UCD		NCD		CD	
	
	UPGMA	NJ	UPGMA	NJ	UPGMA	NJ
Gzip	0.8500	0.9030	0.8500	0.7600	0.9030	0.7600
Bzip2	0.8585	0.6970	0.8265	0.7236	0.9030	0.7462
PPMd16	0.9030	0.7827	0.9030	0.7600	0.9030	0.7827
PPMd8	0.9030	0.8407	0.9030	0.7600	0.5501	0.5456
PPMd4	0.9030	0.7069	0.9030	0.7600	0.5389	0.5517
PPMd2	0.8500	0.7069	0.8500	0.7069	0.5449	0.5386
Huffman	0.8188	0.7609	0.8645	0.7609	0.8161	0.7066
Ac fast	0.8392	0.6770	0.8392	0.6770	0.8410	0.7324
Rc fast	0.8706	0.7275	0.8706	0.7275	0.8734	0.7268
Ac med.	0.8518	0.6718	0.8518	0.6718	0.8584	0.7288
Rc med.	0.8780	0.7674	0.8466	0.6825	0.7936	0.6621
Ac slow	0.8518	0.7009	0.8392	0.7009	0.8584	0.7268
Rc slow	0.9030	0.7357	0.9030	0.7188	0.8734	0.7288
BwtRleHuff	0.8319	0.6970	0.8585	0.8442	0.7840	0.7440
BwtMtfRleHuff	0.8501	0.8355	0.8253	0.6770	0.7971	0.6692
BwtRleAc fast	0.8706	0.7008	0.8706	0.8026	0.9030	0.7515
BwtMtfRleAc fast	0.8382	0.7217	0.8126	0.7336	0.8706	0.8001
BwtRleRc fast	0.8706	0.7343	0.8706	0.6889	0.9030	0.7950
BwtMtfRleRc fast	0.8500	0.7217	0.8500	0.7217	0.8500	0.7515
BwtRleRc med.	0.8500	0.7074	0.8706	0.7336	0.9066	0.8762
BwtMtfRleRc med.	0.8252	0.6951	0.8252	0.6951	0.8252	0.6657
BwtRleRc slow	0.9030	0.7357	0.8585	0.7297	0.9030	0.7084
BwtMtfRleRc slow	0.8706	0.7480	0.8126	0.7910	0.8706	0.6825
BwtWavelet	0.8706	0.6887	0.8706	0.7993	0.8647	0.7066

Experimental results for the CK-36-SEQ data set, with the UCD (left), NCD (middle), and CD (right) distance. For each compression algorithm, we report the F-measure for both UPGMA and NJ methods.

**Table 4:** SP-86-PDB

SP-86-PDB	UCD		NCD		CD	
	
	UPGMA	NJ	UPGMA	NJ	UPGMA	NJ
Gzip	0.5372	0.5265	0.5450	0.5265	0.5372	0.5265
Bzip2	0.5411	0.5265	0.5400	0.5265	0.5440	0.5390
PPMd16	0.5367	0.5265	0.5468	0.5265	0.5477	0.5392
PPMd8	0.5346	0.5265	0.5384	0.5265	0.5477	0.5392
PPMd4	0.5346	0.5265	0.5371	0.5402	0.5388	0.5365
PPMd2	0.5377	0.5265	0.5528	0.5265	0.5439	0.5375
Huffman	0.5335	0.5265	0.5415	0.5265	0.5406	0.5265
Ac fast	0.5399	0.5265	0.5403	0.5265	0.5413	0.5265
Rc fast	0.5399	0.5389	0.5401	0.5299	0.5438	0.5265
Ac med.	0.5407	0.5355	0.5355	0.5276	0.5438	0.5265
Rc med.	0.5407	0.5265	0.5343	0.5317	0.5407	0.5265
Ac slow	0.5407	0.5291	0.5418	0.5265	0.5382	0.5265
Rc slow	0.5407	0.5332	0.5461	0.5283	0.5340	0.5265
BwtRleHuff	0.5376	0.5317	0.5429	0.5269	0.5487	0.5290
BwtMtfRleHuff	0.5374	0.5317	0.5409	0.5359	0.5487	0.5265
BwtRleAc fast	0.5386	0.5366	0.5593	0.5453	0.5481	0.5265
BwtMtfRleAc fast	0.5386	0.5265	0.5486	0.5302	0.5481	0.5265
BwtRleRc fast	0.5386	0.5265	0.5468	0.5265	0.5481	0.5265
BwtMtfRleRc fast	0.5364	0.5356	0.5478	0.5265	0.5481	0.5265
BwtRleRc med.	0.5376	0.5265	0.5439	0.5265	0.5481	0.5265
BwtMtfRleRc med.	0.5351	0.5265	0.5422	0.5265	0.5481	0.5265
BwtRleRc slow	0.5376	0.5265	0.5466	0.5265	0.5540	0.5302
BwtMtfRleRc slow	0.5376	0.5265	0.5426	0.5341	0.5442	0.5291
BwtWavelet	0.5376	0.5265	0.5376	0.5265	0.5449	0.5351

Experimental results for the SP-86-PDB data set, with the UCD (left), NCD (middle), and CD (right) distance. For each compression algorithm, we report the F-measure for both UPGMA and NJ methods.

**Table 5:** SP-86-ATOM

SP-86-ATOM	UCD		NCD		CD	
	
	UPGMA	NJ	UPGMA	NJ	UPGMA	NJ
Gzip	0.5349	0.5337	0.5376	0.5328	0.5338	0.5396
Bzip2	0.5779	0.5510	0.5779	0.5510	0.5632	0.5472
PPMd16	0.5425	0.5460	0.5361	0.5265	0.5567	0.5265
PPMd8	0.5550	0.5550	0.5443	0.5569	0.5562	0.5550
PPMd4	0.5454	0.5459	0.5394	0.5571	0.5482	0.5472
PPMd2	0.5348	0.5297	0.5412	0.5265	0.5418	0.5278
Huffman	0.5303	0.5265	0.5303	0.5265	0.5331	0.5365
Ac fast	0.5553	0.5385	0.5625	0.5524	0.5645	0.5413
Rc fast	0.5587	0.5477	0.5626	0.5389	0.5602	0.5472
Ac med.	0.5580	0.5493	0.5563	0.5438	0.5627	0.5272
Rc med.	0.5581	0.5583	0.5510	0.5434	0.5534	0.5492
Ac slow	0.5440	0.5314	0.5410	0.5265	0.5471	0.5463
Rc slow	0.5363	0.5265	0.5376	0.5265	0.5489	0.5328
BwtRleHuff	0.5408	0.5390	0.5408	0.5332	0.5557	0.5509
BwtMtfRleHuff	0.5411	0.5438	0.5411	0.5438	0.5420	0.5567
BwtRleAc fast	0.5365	0.5282	0.5365	0.5282	0.5323	0.5363
BwtMtfRleAc fast	0.5775	0.5421	0.5775	0.5421	0.5558	0.5747
BwtRleRc fast	0.5317	0.5362	0.5365	0.5462	0.5397	0.5265
BwtMtfRleRc fast	0.5791	0.5421	0.5791	0.5609	0.5558	0.5550
BwtRleRc med.	0.5338	0.5265	0.5338	0.5284	0.5340	0.5317
BwtMtfRleRc med.	0.5390	0.5550	0.5390	0.5550	0.5495	0.5405
BwtRleRc slow	0.5350	0.5385	0.5385	0.5419	0.5415	0.5415
BwtMtfRleRc slow	0.5338	0.5354	0.5338	0.5354	0.5420	0.5694
BwtWavelet	0.5362	0.5344	0.5362	0.5368	0.5339	0.5265

Experimental results for the SP-86-ATOM data set, with the UCD (left), NCD (middle), and CD (right) distance. For each compression algorithm, we report the F-measure for both UPGMA and NJ methods.

**Table 6:** AA-15-DNA

AA-15-DNA	UCD		NCD		CD	
	
	UPGMA	NJ	UPGMA	NJ	UPGMA	NJ
Gzip	4	5	6	9	6	9
Bzip2	6	5	6	5	6	5
PPMd16	4	3	4	3	4	5
PPMd8	4	5	4	5	4	5
PPMd4	8	9	10	13	8	13
PPMd2	24	23	24	23	24	23
Gencompress	4	3	4	3	4	5
Huffman	22	21	22	21	22	23
Ac fast	24	21	22	21	24	23
Rc fast	24	23	22	21	24	21
Ac med.	18	23	24	21	22	21
Rc med.	24	23	22	19	24	21
Ac slow	24	15	16	15	16	17
Rc slow	18	17	14	17	12	17
BwtRleHuff	4	5	4	5	4	5
BwtMtfRleHuff	4	5	4	5	4	5
BwtRleAc fast	6	5	6	5	6	5
BwtMtfRleAc fast	4	5	4	5	4	5
BwtRleRc fast	6	5	6	5	6	5
BwtMtfRleRc fast	4	5	4	5	4	5
BwtRleRc med.	6	5	6	5	6	5
BwtMtfRleRc med.	4	5	4	5	4	5
BwtRleRc slow	6	5	6	5	6	5
BwtMtfRleRc slow	4	5	4	5	4	5
BwtWavelet	6	5	6	5	6	5

Experimental results for the AA-15-DNA data set, with the UCD (left), NCD (middle), and CD (right) distance. For each compression algorithm, we report the partition distance for both UPGMA and NJ methods. Since the data set contains 15 species, the partition distance ranges from 0 to 50 in this case.

**Table 7:** Alignment based results

	UPGMA	NJ
salign-CK-36-PDB-BLOSUM62-local	0.9849	0.7677
salign-CK-36-PDB-PAM120-global	0.9533	0.8556
salign-SP-86-PDB-BLOSUM62-local	0.5391	0.5481
salign-SP-86-PDB-PAM120-global	0.5488	0.5491
cor-word-1-CK-36-PDB	0.5600	0.5718
cor-word-2-CK-36-PDB	0.5837	0.5837
cor-word-3-CK-36-PDB	0.5117	0.5616
cor-word-1-SP-86-PDB	0.5447	0.5356
cor-word-2-SP-86-PDB	0.5319	0.5265
cor-word-3-SP-86-PDB	0.5392	0.5292

Experimental results for the CK-36-PDB and the SP-86-PDB data sets, with different alternative methods to build distance matrices, that is, global and local alignments as well as *k*-tuple statistics and correlations. For each distance matrix associated to a method, we report the F-measure for both UPGMA and NJ methods.

The following conclusions can be drawn from the experiments, with reference to the open questions posed in the Background section:

1) **Question (A)**. In agreement with the ROC analysis, UCD and NCD are virtually indistinguishable. That is, once we have fixed the data set and compressor, the value of the F-measure or partition distance is quite close for UCD and NCD and in most cases better than CD.

2) **Question (B) **UPGMA seems to take better advantage of the USM methodology with respect to NJ. Indeed, given a compressor, the values of the F-measure obtained via UPGMA is in most cases better than those obtained with NJ, by as much as 14 %, on the different representations of the CK-36 data set. On the SK-86 data sets, all compressors and classification algorithms did poorly. Analogous poor performance was obtained with the use of standard techniques, again indicating (see ROC analysis) that SK-86 proteins may be difficult to classify. On the AA-15-DNA data set, once the compressor is fixed, the difference in value of the partition distance is very limited (plus/minus 1) in most cases and large (a maximum of 7) in a few others. It is also worth pointing out that the best performing compression algorithms, with UPGMA and NJ, reach excellent values of the F-measure and partition distance (very close to a perfect classification) on the CK-36 data sets and on the AA-15-DNA data set.

3) **Question (C)**. The results of the ROC analysis are largely confirmed on the CK-36 and SK-86 data sets. In particular, the difference in performance, as measured by the F-measure, between "with memory" compressors and memoryless ones is of some significance only on the CK-36-PDB data set. As for the AA-15-DNA data set, there are a few things worth noting. Gencompress, the best performing compression algorithm for DNA sequences, is the best performer on that data set together with PPMd. However, PPMd is 10 times faster in compression speed (see Table 8) and twice as fast in decompression speed. Moreover, the difference between "with memory" and memoryless algorithms is substantial and there is, in fact, a clear separation in terms of the partition distance values for those two classes of algorithms. So, those results, together with the ones on the other data sets, indicate that "with memory" compressors are substantially better than memoryless compressors only on data sets where there is enough "structure", a property not shared by many data sets of biological relevance.

**Table 8:** Compression ratio

	CK-36-PDB	CK-36-REL	CK-36-SEQ	SP-86-ATOM	SP-86-PDB	AA-15-DNA
Gzip	6.434	13.173	21.271	2.133	6.214	2.415
Bzip2	6.696	16.104	26.808	1.503	6.522	2.197
PPMd16	6.846	13.303	22.996	1.463	6.641	2.108
PPMd8	6.846	13.303	22.996	1.422	6.641	2.109
PPMd4	6.846	13.316	23.043	1.565	6.641	2.047
PPMd2	6.828	13.310	22.996	2.045	6.627	1.966
Huffman	6.307	14.356	25.867	3.590	6.095	2.152
Ac fast	6.991	16.903	28.847	3.573	6.711	1.951
Rc fast	6.952	17.170	30.337	3.581	6.679	1.955
Ac med.	6.650	15.682	27.718	3.533	6.411	1.951
Rc med.	6.631	15.929	29.161	3.536	6.391	1.956
Ac slow	6.350	14.551	26.745	3.542	6.140	1.955
Rc slow	6.364	14.954	28.298	3.545	6.153	1.958
BwtRleHuff	6.580	15.402	28.298	1.849	6.355	2.271
BwtMtfRleHuff	6.589	15.064	27.796	1.644	6.313	2.138
BwtRleAc fast	7.300	18.002	31.592	1.557	6.989	2.141
BwtMtfRleAc fast	7.393	17.345	30.933	1.491	7.033	2.037
BwtRleRc fast	7.266	18.281	33.224	1.558	6.962	2.145
BwtMtfRleRc fast	7.360	17.534	32.376	1.493	7.007	2.041
BwtRleRc med.	6.932	16.981	31.922	1.677	6.667	2.142
BwtMtfRleRc med.	7.025	16.455	31.294	1.561	6.715	2.035
BwtRleRc slow	6.654	15.961	30.824	1.793	6.415	2.142
BwtMtfRleRc slow	6.755	15.669	30.478	1.614	6.473	2.034
BwtWavelet	6.913	15.019	27.686	1.607	6.734	2.188
Gencompress	-	-	-	-	-	1.933

Average compression ratio, in bits per symbol, of the tested algorithms for the six data sets.

4) **Question (D)**. The results of the AUC analysis are largely confirmed here, again showing that the USM methodology has the same performance and limitations as more standard methods. In particular, PPMd together with UCD and UPGMA gives a value of the F-measure within 2% of the value obtained with standard methods. Again, this seems to indicate that the main advantage of the USM methodology with respect to existing ones is its scalability with data set size.

### Compression performance

For completeness, we also report compression ratios on all data sets (Table 8) as well as compression and decompression times on some relevant data sets (Table 9).

**Table 9:** Compression and decompresson speed

	Average compression speed		Average decompression speed	
	
	SP-86-ATOM	AA-15-DNA	SP-86-ATOM	AA-15-DNA
Gzip	0.14	0.40	0.03	0.06
Bzip2	0.26	0.51	0.09	0.16
PPMd16	0.57	0.67	0.61	0.80
PPMd8	0.46	0.65	0.50	0.76
PPMd4	0.34	0.47	0.36	0.53
PPMd2	0.31	0.51	0.33	0.53
Huffman	0.11	0.14	0.29	0.30
Ac fast	0.27	0.22	0.44	0.33
Rc fast	0.17	0.16	0.35	0.35
Ac med.	0.26	0.21	0.44	0.44
Rc med.	0.16	0.19	0.35	0.35
Ac slow	0.26	0.21	0.44	0.43
Rc slow	0.16	0.16	0.35	0.36
BwtRleHuff	0.23	0.43	0.27	0.31
BwtMtfRleHuff	0.23	0.36	0.28	0.32
BwtRleAc fast	0.28	0.38	0.35	0.46
BwtMtfRleAc fast	0.29	0.43	0.35	0.39
BwtRleRc fast	0.23	0.35	0.30	0.36
BwtMtfRleRc fast	0.24	0.37	0.31	0.35
BwtRleRc med.	0.23	0.36	0.30	0.31
BwtMtfRleRc med.	0.24	0.40	0.30	0.35
BwtRleRc slow	0.24	0.42	0.30	0.30
BwtMtfRleRc slow	0.24	0.42	0.31	0.32
BwtWavelet	0.25	0.39	0.43	0.31
Gencompress	-	7.00	-	1.54

Average compression (left) and decompression (right) speed, in *μ*-seconds per input symbol, for the SP-86-ATOM and AA-15-DNA data sets.

## Conclusion

Prior to this research, the USM methodology was perceived as adequate for analysis of biological data mainly because of its flexibility and scalability with data set size. In particular, it would be applicable to any biological data in textual format. That is, it would work well on data sets not necessarily consisting of genomic or proteomic sequences and even with large data sets. Moreover, only the best compression algorithms were recommended for use with the methodology, based on the intuitively appealing explanation that the better the compression guaranteed by a program, the better classification it would guarantee when used with USM. As the results in Table 8 show, memoryless compressors can guarantee compression results comparable to the ones of "with memory" compressors on biological data, even if they are, in general, bad compressors. So, they cannot be dismissed based only on that intuition, even more so since they are fast and use very little main memory. Our study adds to the state-of-the-art the following methodological conclusions and a recipe to use USM on biological data sets.

The USM methodology is worth using, even on data sets of size small enough to be processed by standard methods, including the ones based on alignments. It has the same advantages and limitations of the standard methods, i.e., data sets that can be classified well and others that are difficult to classify. Given that no similarity or dissimilarity measure is likely to be general enough to handle well all data sets, the USM methodology is a valid alternative to existing techniques. Moreover, because of their speed (see Table 9) and low memory requirements, memoryless compressors should be dismissed as not worthy only when the data sets have enough structure-something that should be evaluated using domain knowledge before applying the methodology to a data set.

In general, one of UCD or NCD should be used, in conjunction with UPGMA. As for compression algorithms, when very little is known about whether the data set has structure or not, PPMd and Gencompress are the algorithms likely to give the best results. When speed is important, Gzip is a valid alternative to both of them.

## Methods

### Kolmogorov Complexity and Information Theory: the Universal Similarity Metric and its compression-based approximations

The *conditional Kolmogorov complexity K*(*x*|*y*) of two strings *x *and *y *is the length of the shortest binary program *P *that computes *x *with input *y *[8,40]. Thus, *K*(*x*|*y*) represents the minimal amount of information required to generate *x *by any effective computation when *y *is given as an input to the computation. The *Kolmogorov complexity K *(*x*) of a string *x *is defined as *K*(*x*|*λ*), where *λ *stands for the empty string. Given a string *x*, let x* denote the shortest binary program that produces *x *on an empty input; if there is more than one shortest program, x* is the first one in enumeration order. The *Kolmogorov complexity K *(*x, y*) of a pair of objects *x *and *y *is the length of the shortest binary program that produces *x *and *y *and a way to tell them apart. It is then possible to define the information distance *ID *(*x, y*) between two objects satisfying the following identity, up to logarithmic additive terms:

*ID *(*x, y*) = max {*K *(*x*|*y*), *K *(*y*|*x*)}

Equation (1) is the Kolmogorov complexity of describing object *x*, given *y *and vice versa. It can be shown to be a proper metric [41] and therefore, a distance function for strings. A major further advancement in Kolmogorov complexity-based distance functions has been obtained in [7], where

USM(x,y)=max⁡{K(x|y∗),K(y|x∗)}max⁡{K(x),K(y)}
 MathType@MTEF@5@5@+=feaafiart1ev1aaatCvAUfKttLearuWrP9MDH5MBPbIqV92AaeXatLxBI9gBaebbnrfifHhDYfgasaacH8akY=wiFfYdH8Gipec8Eeeu0xXdbba9frFj0=OqFfea0dXdd9vqai=hGuQ8kuc9pgc9s8qqaq=dirpe0xb9q8qiLsFr0=vr0=vr0dc8meaabaqaciaacaGaaeqabaqabeGadaaakeaacqWGvbqvcqWGtbWucqWGnbqtcqGGOaakcqWG4baEcqGGSaalcqWG5bqEcqGGPaqkcqGH9aqpdaWcaaqaaiGbc2gaTjabcggaHjabcIha4naacmqabaGaem4saSKaeiikaGIaemiEaGNaeiiFaWNaemyEaK3aaWbaaSqabeaacqGHxiIkaaGccqGGPaqkcqGGSaalcqWGlbWscqGGOaakcqWG5bqEcqGG8baFcqWG4baEdaahaaWcbeqaaiabgEHiQaaakiabcMcaPaGaay5Eaiaaw2haaaqaaiGbc2gaTjabcggaHjabcIha4naacmqabaGaem4saSKaeiikaGIaemiEaGNaeiykaKIaeiilaWIaem4saSKaeiikaGIaemyEaKNaeiykaKcacaGL7bGaayzFaaaaaaaa@5EA9@

has been defined and properly denoted as the universal similarity metric. In fact, it is a metric, it is normalized and it is universal: a lower bound to, and therefore a refinement of, any distance function that one can define and compute.

It is well known that there is a relationship between Kolmogorov complexity of sequences and Shannon information theory [42]: the expected Kolmogorov complexity of a sequence *x *is asymptotically close to the entropy of the information source emitting *x*.

Such a fact is of great use in defining workable distance and similarity functions stemming from the theoretic setting outlined above. Indeed, Kolmogorov complexities are non-computable in the Turing sense, so the universal similarity metric must be approximated, via the entropy of the information source emitting *x*. However, it is very hard to infer the information source from the data. So, in order to approximate *K *(*x*), one resorts to *compressive estimates of entropy*.

Let *C *be a compression algorithm and *C *(*x*) (usually a binary string) its output on a string *x*. Let |*C *(*x*)|/|*x*| be its *compression rate*, i.e., the ratio of the lengths of the two strings. Usually, the compression rate of good compressors approaches the entropy of the information source emitting *x *[42]. While entropy establishes a lower bound on compression rates, it is not straightforward to measure entropy itself, as already pointed out. One empirical method inverts the relationship and estimates entropy by applying a provably good compressor to a sufficiently long, representative string. That is, the compression rate becomes a *compressive estimate of entropy*.

In conclusion, we must be satisfied by approximating *K *(*x*) by the length |*C *(*x*)|. Furthermore, since *K *(*x, y*) = *K *(*xy*) up to additive logarithmic precision [7], *K *(*x, y*) can be likewise approximated by the length |*C *(*xy*)| of the compression of the concatenation of *x *and *y*. Finally, and since *K *(*x, y*) = *K *(*x*) + *K *(*y*|x*) = *K *(*y*) + *K *(*x*|y*), again up to constant additive precision [8], the conditional complexity *K *(*x*|y*) is the limit approximation of |*C *(*xy*)| - |*C *(*y*)|, and *K *(*y*|x*) is the limit approximation of |*C *(*yx*)| - |*C *(*x*)|. This gives us our first dissimilarity function:

UCD(x,y)=max⁡{|C(xy)|−|C(x)|,|C(yx)|−|C(y)|}max⁡{|C(x)|,|C(y)|}
 MathType@MTEF@5@5@+=feaafiart1ev1aaatCvAUfKttLearuWrP9MDH5MBPbIqV92AaeXatLxBI9gBaebbnrfifHhDYfgasaacH8akY=wiFfYdH8Gipec8Eeeu0xXdbba9frFj0=OqFfea0dXdd9vqai=hGuQ8kuc9pgc9s8qqaq=dirpe0xb9q8qiLsFr0=vr0=vr0dc8meaabaqaciaacaGaaeqabaqabeGadaaakeaacqWGvbqvcqWGdbWqcqWGebarcqGGOaakcqWG4baEcqGGSaalcqWG5bqEcqGGPaqkcqGH9aqpdaWcaaqaaiGbc2gaTjabcggaHjabcIha4naacmqabaGaeiiFaWNaem4qamKaeiikaGIaemiEaGNaemyEaKNaeiykaKIaeiiFaWNaeyOeI0IaeiiFaWNaem4qamKaeiikaGIaemiEaGNaeiykaKIaeiiFaWNaeiilaWIaeiiFaWNaem4qamKaeiikaGIaemyEaKNaemiEaGNaeiykaKIaeiiFaWNaeyOeI0IaeiiFaWNaem4qamKaeiikaGIaemyEaKNaeiykaKIaeiiFaWhacaGL7bGaayzFaaaabaGagiyBa0MaeiyyaeMaeiiEaG3aaiWabeaacqGG8baFcqWGdbWqcqGGOaakcqWG4baEcqGGPaqkcqGG8baFcqGGSaalcqGG8baFcqWGdbWqcqGGOaakcqWG5bqEcqGGPaqkcqGG8baFaiaawUhacaGL9baaaaaaaa@753B@

Furthermore, the authors of the USM methodology have devised compressive estimates of it: Namely,

NCD1(x,y)=|C(xy)|−min⁡{|C(x)|,|C(y)|}max⁡{|C(x)|,|C(y)|}
 MathType@MTEF@5@5@+=feaafiart1ev1aaatCvAUfKttLearuWrP9MDH5MBPbIqV92AaeXatLxBI9gBaebbnrfifHhDYfgasaacH8akY=wiFfYdH8Gipec8Eeeu0xXdbba9frFj0=OqFfea0dXdd9vqai=hGuQ8kuc9pgc9s8qqaq=dirpe0xb9q8qiLsFr0=vr0=vr0dc8meaabaqaciaacaGaaeqabaqabeGadaaakeaacqWGobGtcqWGdbWqcqWGebardaWgaaWcbaGaeGymaedabeaakiabcIcaOiabdIha4jabcYcaSiabdMha5jabcMcaPiabg2da9maalaaabaGaeiiFaWNaem4qamKaeiikaGIaemiEaGNaemyEaKNaeiykaKIaeiiFaWNaeyOeI0IagiyBa0MaeiyAaKMaeiOBa42aaiWabeaacqGG8baFcqWGdbWqcqGGOaakcqWG4baEcqGGPaqkcqGG8baFcqGGSaalcqGG8baFcqWGdbWqcqGGOaakcqWG5bqEcqGGPaqkcqGG8baFaiaawUhacaGL9baaaeaacyGGTbqBcqGGHbqycqGG4baEdaGadeqaaiabcYha8jabdoeadjabcIcaOiabdIha4jabcMcaPiabcYha8jabcYcaSiabcYha8jabdoeadjabcIcaOiabdMha5jabcMcaPiabcYha8bGaay5Eaiaaw2haaaaaaaa@6CAD@

Based on it, we consider

*NCD *(*x*, *y*) = min {*NCD*_1 _(*x*, *y*), *NCD*_1 _(*y*, *x*)}

Notice that this is a slight variation with respect to the original definition to make the function symmetric. Finally, in the realm of data mining and as an approximation of USM and independently in table compression applications, the following dissimilarity function was proposed:

CD(x,y)=min⁡{|C(xy)|,|C(yx)|,|C(x)|+|C(y)|}|C(x)|+|C(y)|
 MathType@MTEF@5@5@+=feaafiart1ev1aaatCvAUfKttLearuWrP9MDH5MBPbIqV92AaeXatLxBI9gBaebbnrfifHhDYfgasaacH8akY=wiFfYdH8Gipec8Eeeu0xXdbba9frFj0=OqFfea0dXdd9vqai=hGuQ8kuc9pgc9s8qqaq=dirpe0xb9q8qiLsFr0=vr0=vr0dc8meaabaqaciaacaGaaeqabaqabeGadaaakeaacqWGdbWqcqWGebarcqGGOaakcqWG4baEcqGGSaalcqWG5bqEcqGGPaqkcqGH9aqpdaWcaaqaaiGbc2gaTjabcMgaPjabc6gaUnaacmqabaGaeiiFaWNaem4qamKaeiikaGIaemiEaGNaemyEaKNaeiykaKIaeiiFaWNaeiilaWIaeiiFaWNaem4qamKaeiikaGIaemyEaKNaemiEaGNaeiykaKIaeiiFaWNaeiilaWIaeiiFaWNaem4qamKaeiikaGIaemiEaGNaeiykaKIaeiiFaWNaey4kaSIaeiiFaWNaem4qamKaeiikaGIaemyEaKNaeiykaKIaeiiFaWhacaGL7bGaayzFaaaabaGaeiiFaWNaem4qamKaeiikaGIaemiEaGNaeiykaKIaeiiFaWNaey4kaSIaeiiFaWNaem4qamKaeiikaGIaemyEaKNaeiykaKIaeiiFaWhaaaaa@6D96@

### Data sets

The Chew-Kedem data set consists of the following proteins:

**alpha-beta **1aa900, 1chrA1, 1ct9A1, 1gnp00, 1qraA0, 2mnr01, 4enl01, 5p2100, 6q21A0, 6xia00.

**mainly beta **1cd800, 1cdb00, 1ci5A0, 1hnf01, 1neu00, 1qa9A0, 1qfoA0.

**mainly alpha **1ash00, 1babA0, 1babB0, 1cnpA0, 1eca00, 1flp00, 1hlb00, 1hlm00, 1ithA0, 1jhgA0, 1lh200, 1mba00, 1myt00, 2hbg00, 2lhb00, 2vhb00, 2vhbA0, 3sdhA0, 5mbn00.

Protein chain 2vhb00 appears twice in this data set, as 2vhb00 and 2vhbA0, in order to test whether the two chains are detected by compression-based classification methods to be identical (and thus clustered together) or not.

The Sierk-Pearson protein data set is as follows:

**alpha-beta **1a1mA1, 1a2vA2, 1akn00, 1aqzB0, 1asyA2, 1atiA2, 1auq00, 1ax4A1, 1b0pA6, 1b2rA2, 1bcg00, 1bcmA1, 1bf5A4, 1bkcE0, 1bp7A0, 1c4kA2, 1cd2A0, 1cdg01, 1d0nA4, 1d4oA0, 1d7oA0, 1doi00, 1dy0A0, 1e2kB0, 1eccA1, 1fbnA0, 1gsoA3, 1mpyA2, 1obr00, 1p3801, 1pty00, 1qb7A0, 1qmvA0, 1urnA0, 1zfjA0, 2acy00, 2drpA1, 2nmtA2, 2reb01, 4mdhA2.

**mainly beta **1a8d02, 1a8h02, 1aozA3, 1b8mB0, 1bf203, 1bjqB0, 1bqyA2, 1btkB0, 1c1zA5, 1cl7H0, 1d3sA0, 1danU0, 1dsyA0, 1dxmA0, 1et6A2, 1extB1, 1nfiC1, 1nukA0, 1otcA1, 1qdmA2, 1qe6D0, 1qfkL2, 1que01, 1rmg00, 1tmo04, 2tbvC0.

**mainly alpha **1ad600, 1ao6A5, 1bbhA0, 1cnsA1, 1d2zD0, 1dat00, 1e12A0, 1eqzE0, 1gwxA0, 1hgu00, 1hlm00, 1jnk02, 1mmoD0, 1nubA0, 1quuA1, 1repC1, 1sw6A0, 1trrA0, 2hpdA0, 2mtaC0.

The Apostolico data set consists of the mitochondrial DNA complete genome of the following species:

**Laurasiatheria **blue whale (*B. musculus*), finback whale (*B. physalus*), white rhino (*C. simum*), horse (*E. caballus*), gray seal (*H. grypus*), harbor seal (*P. vitulina*).

**Murinae **house mouse (*M. musculus*), rat (*R. norvegicus*).

**Hominoidea **gorilla (*G. gorilla*), human (*H. sapiens*), gibbon (*H. lar*), pigmy chimpanzee (*P. paniscus*), chimpanzee (*P. troglodytes*), sumatran orangutan (*P. pygmaeus abelii*), orangutan (*P. pygmaeus*).

### Algorithms

#### Compression algorithms

The compression algorithms we have used for the computation of the dissimilarity functions defined earlier, together with their parameter setting-a crucial and many times overlooked aspect of data compression-can be broadly divided into four groups. The first group consists of three state-of-the-art tools for general purpose compression.

Gzip and Bzip2 These are the well known compression tools based respectively on the classic Lempel-Ziv algorithm [43] and the bwt (Burrows-Wheeler transform) [44].

PPMd This algorithm, written by Dmitry Shkarin [45,46], is the current state-of-the-art for PPM compression. In our tests we used it with four different context lengths (see below).

The algorithms in the second group are known as order zero-or memoryless-compressors since the codeword for a symbol only depends on its overall frequency (for Huffman Coding) or on its frequency in the already scanned part of the input (for Range and Arithmetic Coding).

Huffman An implementation of the classic two-pass Huffman coding scheme.

Ac Arithmetic coding algorithm as implemented in [47].

Rc Range coding algorithm. Range coding and arithmetic coding are based on similar concepts and achieve similar compression. We used the Range Coding implementation from [48].

The algorithms in the next group are all based on the bwt and have been implemented and tested in [49].

BwtMtfRleHuff and BwtRleHuff. The first of these algorithms consists in computing the bwt followed by Move-to-front encoding, followed by the run-length encoding, followed by Huffman coding (as implemented by algorithm Huffman). The algorithm BwtRleHuff is analogous to Huffman except that the Move-to-front encoding step is omitted.

BwtMtfRleAc and BwtRleAc are analogous respectively to BwtMtfRleHuff and BwtRleHuff, except that in the final step they use Arithmetic Coding (algorithm Ac) instead of Huffman coding.

BwtMtfRleRc and BwtRleRc are analogous respectively to BwtMtfRleHuff and BwtRleHuff, except that in the final step they use Range Coding (algorithm Rc) instead of Huffman Coding.

BwtWavelet computes the bwt of the input and compresses the resulting string using a wavelet tree encoder [50].

Finally, we used an algorithm specially designed for compressing DNA sequences.

Gencompress This is currently the best available algorithm to compress DNA sequences [51]. It makes clever use of approximate occurrences of substrings in DNA sequences to achieve good compression.

#### Range/arithmetic coding variants

The behavior of range and arithmetic coding depends on two parameters: MaxFreq and Increment. The ratio between these two values essentially controls how quickly the coding "adapts" to the new statistics. For range coding we set MaxFreq = 65, 536 (the largest possible value) and we experimented with three different values of Increment. Setting Increment = 256 we get a range coder with FAST adaptation, with Increment = 32 we get a range coder with MEDIUM adaptation and finally, setting Increment = 4 we get a range coder with SLOW adaptation. Similarly for arithmetic coding we set MaxFreq = 16, 383 (the largest possible value) and Increment equal to 64, 8, 1 to achieve respectively FAST, MEDIUM, and SLOW adaptation.

#### PPMd variants

The compressive power of PPMd is strictly related to the length of the contexts which are used for predicting the next symbol. In our experiments, we have used models (contexts) of length 2, 4, 8 and 16, and 256 Mb of working memory. Contexts of length 16 represent PPMd at its maximum strength. In the tables the number beside PPMd indicates the context length used.

#### Alignment and alignment-free algorithms

The alignment algorithms we have used are the global alignment algorithm of [52] with the PAM120 substitution scoring matrix [53], and the local alignment algorithm of [54] with the BLOSUM62 substitution scoring matrix [55], as implemented in the R package pairseqsim [56].

For alignment-free comparison, we have computed the linear correlation coefficient between the 20^*k *^dimensional vectors of *k*-mer frequencies [5,15] for *k *= 1, 2, 3, using the R package seqinr [57].

#### Dissimilarity matrix construction algorithms

We have produced BioPerl scripts that take as input a set of files to be classified and a compression algorithm (together with all of its proper parameter settings) and that return a dissimilarity matrix. In particular, there is a preprocessing script that computes all the needed compression values in order to compute UCD, NCD and CD. In turn, there is a script corresponding to each of them that, given as input the results of the preprocessing step, produces the actual dissimilarity matrix. A detailed description of these scripts is given in the readme file at the supplementary material web site, at [1].

### ROC curves and external measures

A ROC curve [58] is a plot of the true positive rate (the sensitivity) versus the false positive rate (one minus the specificity) for a binary classifier as the discrimination threshold changes. The AUC is a value ranging from 1 for a perfect classification, with 100% sensitivity (all true positives are found) to 0 for the worst possible classification, with 0% sensitivity (no true positive is found). A random classifier has an AUC value around 0.5. When plotted, the better the ROC curve follows the ordinate and then the abscissa line, the better the classification. The ROC curve of a random classifier, when plotted, is close to the diagonal. The *F*-measure [59] takes in input two classifications of objects, that is, two partitions of the same set of objects, and returns a value ranging from 0 for highest dissimilarity to 1 for identical classifications. The partition (or symmetric difference) distance [60,61], takes in input the tree topologies of two alternative classifications of *n *species and returns a value ranging from 0 to 4*n *- 10. It is the number of clades in the two rooted trees that do not match and it is increasing with dissimilarity. When zero, it indicates isomorphic trees.

### Experimental set-up and availability

All the experiments have been performed on a 64-bit AMD Athlon 2.2 GHz processor, 1 GB of main memory running both Windows XP and Linux. All software and data sets involved in our experimentation is available at the supplementary web site [1]. The software is given both as C++ code and Perl scripts and as executable code, tested on Linux (various versions – see supplementary material website), BSD Unix, Mac OS X and Windows operating systems.

## Competing interests

The author(s) declare that they have no competing interests.

## Authors' contributions

RG and GV conceived the method and prepared the manuscript. GV implemented part of the software and performed the ROC analysis and some of the experiments. VG implemented part of the software and performed most of the experiments. PF and GM contributed software for compression methods. All authors contributed to the discussion and have approved the final manuscript.
